# Clinical Characteristics and Treatment Outcomes Among Patients With Gastrointestinal Phytobezoars: A Single-Institution Retrospective Cohort Study in Korea

**DOI:** 10.3389/fsurg.2021.691860

**Published:** 2021-06-24

**Authors:** Songsoo Yang, Min Jeng Cho

**Affiliations:** Department of Surgery, Ulsan University Hospital, University of Ulsan College of Medicine, Ulsan, South Korea

**Keywords:** bezoars, endoscopy, intestinal obstruction, surgery, gastric surgery

## Abstract

**Purpose:** To describe our experience with phytobezoars, evaluate risk factors on treatment, and analyze whether previous gastric surgery affects treatment outcomes.

**Methods:** Medical records of 51 patients with phytobezoars between 2000 and 2019 were retrospectively evaluated. We compared endoscopic and surgical treatment groups and evaluated risk factors using multivariate logistic regression analysis. And we compared patients with and without previous gastric surgery in the surgical treatment group.

**Results:** The median patient age was 62.9 (range: 27–89) years. The endoscopic and surgical treatment groups included 26 (51%) and 25 (49%) patients, respectively. Patients aged ≥65 years, diabetes, and small intestinal phytobezoars were more frequent in the surgical treatment group. Previous gastric surgery (*n* = 16, 31.4%) was the most common predisposing risk factor, but without a significant difference between the groups. Enterotomy was performed for 20 patients (80%), segmental resection was performed for five patients (20%). Five patients (20%) had postoperative complications; there was one death. There were no significant differences in age, preoperative diagnosis, operation method, operative time, or postoperative stay between patients with and without previous gastric surgery, but postoperative complications were significantly more common in patients with previous gastric surgery.

**Conclusions:** Phytobezoar should be suspected early in patients with previous gastric surgery or a specific food intake history. Early diagnosis and treatment are important for avoiding surgical intervention and complications, especially in elderly patients. Surgery is required in most patients with small intestinal phytobezoars, safe removal can be achieved mainly via enterotomy.

## Introduction

Bezoars are defined as aggregates of inedible or undigested material that has been orally ingested. Bezoars are categorized into phytobezoars (undigested food), trichobezoars (hair), lactobezoars (milk protein), and pharmacobezoars (medications) based on their components (1, 2); phytobezoars are the most common. The incidence of phytobezoars varies depending on ethnic and geographic factors and has been correlated with the consumption of persimmons and high fiber-containing foods. In the stomach acid, persimmon tannin may polymerize, and form a conglomerate in which cellulose, hemicelluloses, and proteins accumulate (3–7). Indeed, phytobezoars tend to be more common in regions such as Asia, Spain, Israel, and Turkey (5–7). In addition, poor mastication and medical conditions such as diabetes and hypothyroidism have been described as risk factors for the development of phytobezoars. However, given that it alters the anatomy and physiology of the stomach, previous gastric surgery represents the most common predisposing factor for phytobezoars, with 20–93% of patients reporting a relevant surgical history (5, 8).

Although phytobezoars usually form in the stomach, they can descend through the small bowel and cause mechanical intestinal obstruction. Other symptoms of phytobezoars include abdominal pain, nausea, vomiting, and gastrointestinal bleeding. Early diagnosis and treatment are critical due to the risks of decubitus ulcers, pressure necrosis, perforation, and strangulation (9, 10). Currently, endoscopic procedures and surgical treatments are considered the primary therapeutic options for phytobezoars.

Korean individuals tend to consume a diet rich in high fiber foods and persimmons, and the rate of gastric surgery is high due to the high incidence of gastric cancer in the Korean population (11). In the present study, we aimed to (i) describe our experience with phytobezoars in a Korean population to aid physicians in diagnosing and managing this uncommon condition, (ii) evaluate the effect of risk factors on treatment modalities (i.e., endoscopy and surgery), and (iii) analyze whether previous gastric surgery affects diagnosis and treatment outcomes among patients with phytobezoars.

## Materials and Methods

We performed a computerized search of our clinical database for adult patients (≥18 years of age) treated for bezoars at our institution between October 2000 and December 2019. Patients with trichobezoars and pharmacobezoars and those treated conservatively without endoscopic or surgical intervention were excluded.

We identified 51 patients with phytobezoars, including 3 patients with food bolus bezoars in the endoscopy or operating room. We collected and analyzed data related to demographic characteristics, clinical signs and symptoms, previous medical and surgical history, location and size of bezoars, treatment modality, hospitalization period, and morbidity and mortality rates. The study protocol was approved by the internal review board of our institution (2020-09-033).

For diagnostic purposes, 22 patients underwent endoscopy, 20 patients underwent computed tomography (CT), and 9 patients underwent both endoscopy and CT. Endoscopic treatment was performed by experienced endoscopists. Endoscopic forceps, baskets, or snares were used for fragmentation. Effective dissolution was ensured via drinking, nasogastric lavage, or endoscopic injection of cola if necessary. Surgical treatment involved an open or laparoscopic approach under general anesthesia. Phytobezoars were extracted via gastrotomy or enterotomy. Segmental resection and anastomosis were performed in ischemia with strangulation or large-sized phytobezoars. Laparoscopic assisted enterotomy or bowel resection was performed by locating the bezoar-containing bowel segment via diagnostic laparoscopy and bringing the intestine to the outside through a small incision.

Previous gastric surgeries included vagotomy, pyloroplasty, and gastrectomy (partial or total), all of which can affect acid production and gastric outlet. Patients were classified into two groups based on management modality: an endoscopic treatment group (endoscopic intervention only) and a surgical treatment group (surgery with pre- and post-operative endoscopy). Endoscopic intervention was attempted in all patients judged fit to undergo endoscopic treatment by the endoscopist, and surgical treatment was performed when endoscopic treatment was unsuccessful or when bezoar was not found in the endoscopy. Complications were categorized according Clavien-Dindo classification (12).

Continuous variables (median with range or mean ± standard deviation) were analyzed using independent Student's *t*-tests or Mann-Whitney U-tests, while categorical variables (frequencies and proportions) were analyzed using Pearson χ2 tests or Fisher exact tests. We also evaluated the effects of age (≥65 years old), diabetes, and small intestinal phytobezoars presence on the risk of surgery using multivariate logistic regression analysis. The data were analyzed using the Statistical Program for the Social Sciences (IBM SPSS Statistic Version 24, IBM Inc., Chicago, IL). A *P*-value of < 0.05 was considered statistically significant.

## Results

### Endoscopic vs. Surgical Treatment

The median age of the total cohort of 51 patients (27 male and 24 female) was 62.9 years (range: 27–89 years). The endoscopic and surgical treatment groups included 26 (51%) and 25 (49%) patients, respectively. The clinical characteristics of the two groups are summarized in Table 1. Patients aged ≥65 years and diabetes were more common in the surgical treatment group than in the endoscopic treatment group (*P* = 0.007, *P* = 0.024, respectively). Sixteen patients (31.4%) had previous gastric surgery (Table 2). The interval between gastric operation and phytobezoars detection ranged from 9 months to 30 years. There was no difference in the rate of previous gastric surgery between the endoscopic treatment and surgical treatment groups (*P* = 0.485).

**Table 1:** Clinical characteristics in the endoscopic and surgical treatment groups.

	**Endoscopic treatment group (*n* = 26)**	**Surgical treatment group (*n* = 25)**	***P***
Age (yr)	58.6 ± 12.8	67.4 ± 13.4	0.02
≥65 yr	6 (23.1)	15 (60.0)	0.007
<65 yr	20 (76.9)	10 (40.0)	
Gender M:F	15:11	12:13	0.488
Underlying disease			
Hypertension	4 (15.4)	8 (32.0)	0.199
Diabetes	1 (3.8)	7 (28.0)	0.024
Peptic ulcer disease	1 (3.8)	1 (4.0)	1.0
Persimmon intakes	5 (19.2)	6 (24.0)	0.743
Brown seaweed intakes	2 (7.7)	2 (8.0)	0.632
Other	2 (7.7)^a^	5 (20.0)^b^	0.248
Previous abdominal surgery			
Gastric surgery	7 (43.8)	9 (56.3)	0.485
Other abdominal surgery	1 (3.8)^c^	6 (24.0)^d^	0.05
Symptoms			
Abdominal pain	10 (38.5)	18 (72.0)	0.016
Nausea and vomiting	6 (23.1)	16 (64.0)	0.003
Melena	0 (0)	2 (8.0)	0.235
Abdominal distension	1 (3.8)	10 (40.0)	0.002
Duration of symptoms before admission (d)	24.6 ± 25.3	16.3 ± 24.1	0.239
Location of phytobezoar			<0.001
Stomach	22 (84.6)	1 (4.0)	
Small bowel	4 (15.4)	24 (96.0)	
Diagnostic workup			
CT	4 (15.4)	25 (100.0)	<0.001
Endoscopy	26 (100.0)	5 (20.0)	<0.001
Coexisting gastric ulcers	12 (46.2^e^)	5 (100.0^e^)	<0.001

*Values are presented as mean ± standard deviation (range) or number (%)*.

*CT, computed tomography*.

a *Liver cirrhosis (n = 1), chronic renal disease (n = 1)*.

b *Coronary artery disease (n = 3), arrhythmia (n = 1), liver cirrhosis (n = 1)*.

c *Hysterectomy (n = 1)*.

d *Cholecystectomy (n = 3), hysterectomy (n = 1), appendectomy (n = 1)*.

e *Percentage of gastric ulcers in endoscopic performed patients*.

**Table 2:** Type of previous gastric surgery.

	**Patients (*n*)**	**Cause of operation**
Truncal vagotomy and pyloroplasty	3	Three peptic ulcer
Gastrectomy (distal subtotal) and Billroth I anastomosis	3	Three gastric cancer
Gastrectomy (distal subtotal) and Billroth II anastomosis	2	One gastric cancer/One peptic ulcer
Laparoscopic gastrectomy (distal subtotal) and Billroth I anastomosis	5	Five gastric cancer
Laparoscopic gastrectomy (distal subtotal) and Billroth II anastomosis	2	Two gastric cancer
Truncal vagotomy and gastrectomy (Mckeown operation)	1	One esophageal cancer

In the endoscopic treatment group, 18 patients (69.2%) were treated using mechanical fragmentation only, while the remaining eight (30.8%) were treated using mechanical fragmentation with cola. Phytobezoars were successfully eliminated in a single endoscopic session in 12 patients. Seven patients required two sessions, five patients required three sessions, one patient required six sessions, and one patient required eight sessions.

In the endoscopic treatment group, phytobezoars were usually located in an area proximal to the stomach (88.5%), whereas they were predominantly located in the small intestine in the surgical treatment group (96.0%). Four patients of the surgical treatment group were diagnosed with gastric phytobezoars via endoscopy at other primary clinics, although they did not receive treatment. When these patients visited our emergency department (ED), the phytobezoars had migrated to the small intestine and caused obstruction, thus necessitating surgery. Partial removal was achieved via endoscopy in one patient, who required surgery to remove the remaining portion, which had migrated to the small intestine.

Age ≥65 years and small intestinal phytobezoars were significantly predictive of surgical treatment (odds ratio (OR): 5.850, 70.934, respectively (95% confidence interval (CI): 1.013–33.795, 5.947–846.065, respectively) (Table 3). The logistic regression model was consistent with findings reported by Hosmer and Lemeshow using the goodness-of-fit test (*P* = 0.707).

**Table 3:** Multivariate logistic regression analysis of risk factors effective in patients with surgical treatment.

	**Odds ratios**	**95% CI**	***P***
Age ≥65 years	5.850	1.013–33.795	0.048
Diabetes	7.538	0.504–112.800	0.143
Small intestinal phytobezoars	70.934	5.947–846.065	<0.001

### Surgical Treatment of Phytobezoars

Table 4 shows the characteristics and outcomes of 25 patients who underwent surgery, as well as differences between patients with and without previous gastric surgery. All patients underwent preoperative CT, which identified phytobezoars in 18 patients (72%). Of the seven misdiagnoses, six were interpreted as adhesions or non-specific ileus, while one was interpreted as gastric perforation with an unknown cause.

**Table 4:** Surgical treatment of phytobezoars.

	**Total (*n* = 25)**	**Without previous gastric surgery (*n* = 16)**	**With previous gastric surgery (*n* = 9)**	***P***
Age (yr)	67.4 ± 13.4	68.1 ± 14.3	66.1 ± 12.3	0.726
Gender M:F	12:13	5:11	7:2	0.041
CT				0.205
Diagnostic	18 (72.0)	13 (81.3)	5 (55.6)	
Non-diagnostic	7 (28.0)	3 (18.8)	4 (44.4)	
Size of phytobezoar (cm)	7.7 ± 7.2	11.5 ± 13.3	5.5 ± 2.0	0.092
Location of phytobezoar				0.105
Stomach	1 (4.0)	1 (6.3)	0 (0)	
Jejunum	11 (44.0)	9 (60.0)	2 (22.2)	
Ileum	13 (52.0)	6 (40.0)	7 (77.8)	
Strangulation	2 (8.0)	2 (12.5)	0 (0)	0.520
Operation method				0.312
Enterotomy	20 (80.0)	14 (87.5)	6 (66.7)	
Segmental resection	5 20.0)	2 (12.5)	3 (33.3)	
Laparoscopic assisted	12 (48.0)	7 (43.8)^a^	5 (55.6)	0.688
Operative time (min)	96.6 ± 42.5	100.3 ± 50.6	90.0 ± 23.2	0.571
Hospitalization day (d)	16.4 ± 7.8	14.0 ± 4.5	20.6 ± 10.7	0.111
From admission to operative (d)	5.2 ± 5.1	4.4 ± 4.2	6.7 ± 6.3	0.299
Postoperative stay (d)	11.0 ± 6.3	9.4 ± 3.5	14.0 ± 9.0	0.170
Complication^b^	6 (24.0)	1 (6.3)	5 (55.6)	0.002
Grade I	1	0	1	
Grade II	3	0	3	
Grade III	0	0	0	
Grade IV	1	0	1	
Grade V	1	1	0	

*Values are presented as mean ± standard deviation (range) or number (%)*.

*CT, computed tomography*.

a *Include one patient who required an open conversion due to adhesion from a previous hysterectomy*.

b *Clavien-Dindo classification*.

**Figure 1:**
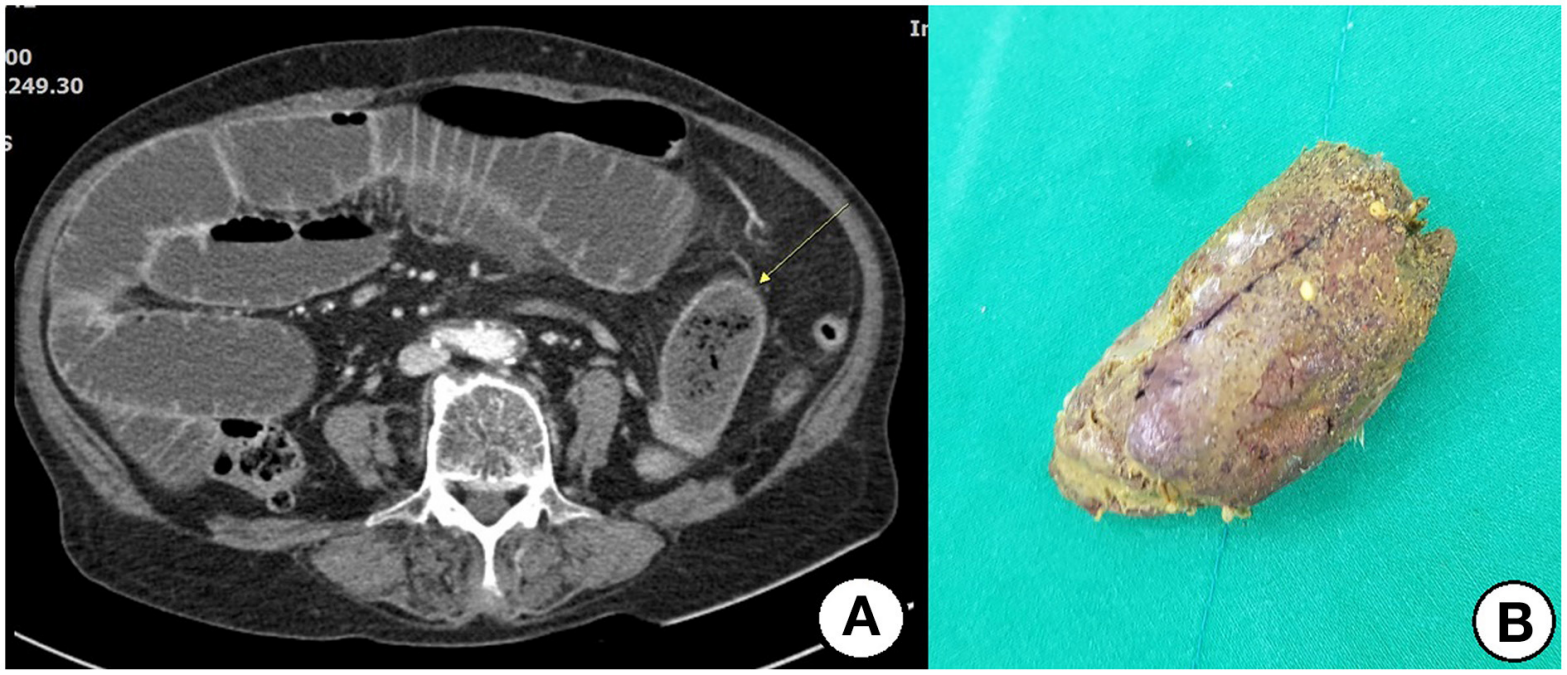
**(A)** Contrast-enhanced CT shows distended small bowel and an ovoid mass (arrow) with mottled gas pattern at the obstruction site. **(B)** Removed phytobezoar.

**Figure 2:**
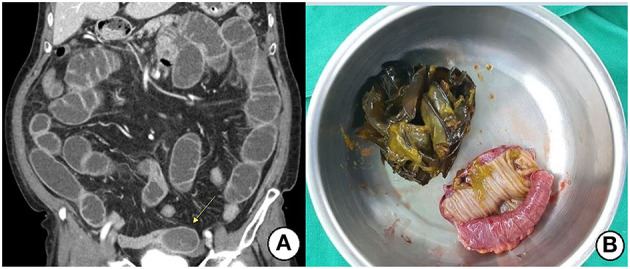
**(A)** Contrast-enhanced CT shows an ovoid heterogeneous mass (arrow) and proximal distended small bowel. **(B)** Segmental resection and anastomosis were performed due to the large-sized seaweeds bolus.

All patients in the surgical group exhibited obstructions with proximally dilated loops. Round phytobezoars of unknown origin were identified in 22 patients (Figure 1). Undigested food bolus bezoars were identified in the remaining three patients (two brown seaweed, one young radish) (Figure 2). Enterotomy was performed for 20 patients (80%), while segmental resection was performed for five patients (20%). There were two cases (8%) of ischemic bowel. Twelve patients (48%) underwent surgery using a laparoscopic assisted technique, three of whom were treated using a single incision port. Conversion was required in one case due to adhesion after the initial laparoscopic attempt.

Postoperative complications developed in five patients (20%): pseudomembranous colitis (PMC) and acute renal failure (ARF) in one patient, intraabdominal abscess and wound infection in one patient, wound infection in one patient, and prolonged ileus in two patients. There was one case of death (2%): a 66-year-old man with diabetes and severe alcoholic liver cirrhosis (Child-Pugh classification Class C) who had visited the ED with septic shock. CT revealed a perforation of unknown cause in the gastric upper body, immediately following which laparotomy was performed. Surgical findings indicated that a phytobezoar of more than 30 cm had filled the entire stomach, and an ulcer had perforated due to the presence of a longstanding phytobezoar. The phytobezoar was removed, and the site of perforation was repaired. However, the patient died on the fourth postoperative day due to multiorgan failure.

We observed no significant differences in age, preoperative diagnosis, operation method, operative time, or postoperative stay between patients with and without previous gastric surgery. Phytobezoars were more frequently located in the jejunum in patients without previous gastric surgery, while they were more frequently located in the ileum in those with previous gastric surgery; however, this difference was not significant (*P* = 0.105). Male gender and postoperative complications were significantly more common in patients with previous gastric surgery than those without (*P* = 0.041, *P* = 0.002, respectively).

## Discussion

The present study investigated the characteristics of phytobezoars in a Korean population. Our results demonstrated that age ≥65 years and small intestinal phytobezoars are significant predictors of surgical treatment. Although the history of gastric surgery was an important predisposing factor associated with phytobezoars, such history did not influence treatment approaches, operation methods, or treatment period. However, the rate of postoperative complications was higher in patients with a history of gastric surgery than in those without.

Phytobezoars are mostly formed of poorly digested fibers, fruit seeds, and pulpy fruits. Large series have indicated that gastric phytobezoars are found in <0.5% of endoscopies (4, 6), and that the rate of small intestinal obstruction secondary to phytobezoars ranges from 2 to 4% (7, 13). Previous gastric surgery, poor mastication, a high fiber diet, and diabetes are known risk factors that facilitate the development of phytobezoars (14, 15). Previously, gastric surgery for peptic ulcer disease had predisposed patients to phytobezoars (14, 16). Vagotomy and/or partial gastrectomy results in a hypoacidic environment and reduced gastric motility, which diminishes the ability of the stomach to break up and digest food. Widening of the gastric outlet via gastroenterostomy or pyloroplasty can easily lead to the passage of a large, non-fragmented bolus to the small intestine, resulting in downstream impaction. However, patients with peptic ulcers are now more commonly treated using conservative strategies (17). Nonetheless, gastric cancer remains the most common cancer in Korea, with an annual incidence rate of 57.9/100,000, and many patients still require gastric surgery (11). In accordance with previous findings, the most common predisposing risk factor identified in our study was previous gastric surgery (31.4% of patients) (7, 8, 14, 17). However, most patients in these other studies had undergone surgical treatment for peptic ulcers (7, 8, 14, 17), whereas 75% of our patients had undergone surgical treatment for gastric cancer. The incidence of phytobezoars formation after gastric surgery ranges from 5 to 12% (5, 8), highlighting the importance of regular evaluations after gastric surgery.

Medical conditions such as diabetes, hypothyroidism, and antacid drug use alter gastric physiology in a similar manner, potentially leading to phytobezoars (18, 19). Dietary factors, chewing habits, and impaired mastication have also been associated with phytobezoars development (20). In particular, phytobezoars are more common in elderly patients due to decreased masticatory function, resulting in reduced fiber breakdown. Traditional Asian diets are high in fiber, and pumpkin, grape skin, plums, raisins, and persimmons are common causes of phytobezoars (5–7). Eleven patients (21.6%) in our study reported a history of persimmon intake. Interestingly, brown seaweed consumption was noted as the cause of phytobezoars in four of our patients (7.8%), which has not been reported previously. Uncooked brown seaweeds are a favorite side dish along the south coast of Korea, including Ulsan. Uncooked brown seaweeds are usually eaten in pieces larger than 5 cm, and it enters the gastrointestinal tract without much fragmentation in the mouth. This may explain the presence of intact brown seaweeds leading to intestinal obstruction in the present study.

We also evaluated the effect of the above-mentioned risk factors on treatment modality. Multivariate logistic regression analysis revealed that age ≥65 years and small intestinal phytobezoars were independent risk factors for surgical treatment. This result emphasizes that early diagnosis and treatment are important for patients with suspected phytobezoars, especially elderly patients.

In accordance with previous findings, most phytobezoars located in the stomach were easy to diagnose and have a high rate of successful removal via endoscopy. In contrast, phytobezoars that had migrated to the small intestine were difficult to diagnose and more likely to require surgery (7, 13, 17, 18). Although all patients in the surgical treatment group underwent CT, only 72% were diagnosed with phytobezoars prior to surgery. Preoperative diagnosis was difficult because the rate of phytobezoar- induced intestinal obstruction was low, and no specific clinical findings separated phytobezoars from other intestinal obstructions (18, 21, 22). Typical phytobezoars in the intestine appear as well-defined, ovoid, or round heterogeneous intraluminal masses with a mottled gas appearance (18, 22), but this does not apply to all phytobezoars as these can be affected by the content and time taken for formation. For example, in our study, lesions such as seaweed did not show a typical appearance on CT. The most common cause of small intestinal obstruction in patients with previous abdominal surgery is postoperative adhesions, for which non-operative treatment strategies are commonly considered (5, 7). Therefore, the clinician's suspicion and careful history investigation are necessary to ensure a fast and accurate diagnosis. Although four patients in the surgical treatment group were diagnosed with gastric phytobezoars via endoscopy at other primary clinics, they did not receive treatment. In these patients, phytobezoars had migrated to the small intestine, causing obstruction and necessitating surgery following arrival in the ED. Therefore, active intervention is essential for patients with gastric phytobezoars, even when endoscopy is performed several times. As observed in the present study, gastric perforation is a serious complication of phytobezoars. Phytobezoars in the stomach may cause bleeding and perforation in patients with coexisting decubitus ulcers, and small intestinal obstruction from phytobezoars can lead to strangulation or perforation if the diagnosis is delayed (9, 18).

Several studies have reported that the most common site for small intestinal phytobezoars is the terminal ileum, which is the narrowest point of the small intestine and has been associated with delayed transit (13, 17, 23). However, phytobezoars rates for the jejunum and ileum were similar in our study. We speculate that patients without previous gastric surgery had occlusions in the upper part of the small intestine due to the presence of larger phytobezoars, while those with previous gastric surgery had phytobezoars located in the ileum, despite a lack of statistical significance (*P* = 0.105). In this study, enterotomy was performed in 20 patients (80%), and segmental resection was required in cases of strangulation or large-sized phytobezoars. We also analyzed the effect of previous gastric surgery on preoperative diagnosis, location, surgical method, and outcome. There were no significant differences in age, location of phytobezoars, operation method, laparoscopic approach, or operative time between patients with and without previous gastric surgery. However, the rate of complications was higher in those with previous gastric surgery than in those without. We speculate that previous surgery may affect adjacent organs during adhesiolysis, leading to complications such as prolonged ileus and intraabdominal abscess.

Recent reports have cited the feasibility of laparoscopy in managing bezoars-induced small intestinal obstruction (24–26). Yau et al. demonstrated that the laparoscopic approach is associated with a significantly shorter operative time, shorter hospital stay, and fewer complications than the conventional open method (26). Laparoscopic surgery for bezoar removal is difficult if there is adhesion due to previous abdominal surgery or insufficient space in the abdominal cavity due to proximal intestinal dilatation. Horesh et al. reported that the conversion rate was relatively high (i.e., 55%) (6). In our study, 12 patients (48%) underwent laparoscopic assisted surgery, and only one patient underwent open conversion. Single incision laparoscopy was performed in three cases. Thus, our findings indicate that laparoscopic surgery is safe and possible regardless of previous surgical history.

Our study has several limitations. First, its retrospective design and incomplete medical records may have contributed to selection bias. Furthermore, we could not evaluate eating habits or chewing problems caused by dental issues because relevant responses were not routinely included in medical records. Second, since patients underwent different types of previous gastric surgery, we could not evaluate the effect of each type on phytobezoars outcomes, nor could we evaluate the influence of other types of previous abdominal surgery. Finally, given its single center design, our study is also limited by its small sample size. Nevertheless, based on our experience, our findings highlight the need for early suspicion of phytobezoars in patients with previous gastric surgery or a specific history of food intake (e.g., diets high in fiber, persimmons, and brown seaweeds). In addition, regular endoscopy after gastric surgery including procedures for gastric cancer may aid in the early detection of phytobezoars in the Korean population.

In conclusion, early diagnosis and rapid treatment of phytobezoars are important in avoiding surgical intervention and complications, especially in elderly patients. Although surgical treatment may be required in most patients with small intestinal phytobezoars, safe removal can be achieved mainly via enterotomy. Patients with a history of gastric surgery should again be monitored for complications following such procedures.

## Data Availability Statement

The raw data supporting the conclusions of this article will be made available by the authors, without undue reservation.

## Author Contributions

MC: study conception and design, critical revision, and drafting of the manuscript. SY and MC: analysis, data interpretation, and data acquisition. All authors contributed to the article and approved the submitted version.

## Conflict of Interest

The authors declare that the research was conducted in the absence of any commercial or financial relationships that could be construed as a potential conflict of interest.
